# A Systematic Framework for Optimizing a Sweeping Gas Membrane Distillation (SGMD)

**DOI:** 10.3390/membranes10100254

**Published:** 2020-09-24

**Authors:** Nawras N. Safi, Salah. S. Ibrahim, Nasser Zouli, Hasan Shaker Majdi, Qusay F. Alsalhy, Enrico Drioli, Alberto Figoli

**Affiliations:** 1 Membrane Technology Research Unit, Chemical Engineering Department, University of Technology, Alsinaa Street 52, Baghdad 10066, Iraq; nawras.nabeel@yahoo.com (N.N.S.); salah.s.ibrahim@uotechnology.edu.iq (S.S.I.); 2 Department of Chemical Engineering, Jazan University, P.O. Box 706, Jazan 45142, Saudi Arabia; nizouli@jazanu.edu.sa; 3 Department of Chemical Engineering and Petroleum Industries, AlMustaqbal University College, Babylon 51001, Iraq; hasanshker1@gmail.com; 4 Institute on Membrane Technology, National Research Council (ITM-CNR), Rende 87030, CS, Italy; e.drioli@itm.cnr.it (E.D.); a.figoli@itm.cnr.it (A.F.)

**Keywords:** taguchi method, sweeping gas membrane distillation (SGMD), desalination, membrane performance, optimization

## Abstract

The present work has undertaken a meticulous glance on optimizing the performance of an SGMD configuration utilized a porous poly (vinylidene fluoride-co-hexafluoropropylene) (PVDF-*co*-HFP) membrane. This was carried out by conducting a systematic framework for investigating and optimizing the pertinent parameters such as sweeping gas flow rate, feed temperature, feed concentration and feed flow rate on the permeate flux. For this purpose, the Taguchi method and design of experiment techniques were harnessed to statistically determine optimum operational conditions. Besides that, a comprehensive surface and permeation characterization was conducted against the hand-made membranes. Results showcased that the membrane performance was ultimately controlled by the feed temperature and was nearly (~680) % higher when the temperature raised from 45 to 65 °C. Also, to a lesser extent, the system was dominated by the feed flow rate. As the adopted feed flow rate increases (from 0.2 to 0.6 L/min), around 47.5% increment was bestowed on water permeability characteristics. In contra, 34.5% flux decline was witnessed when higher saline feed concentration (100 g/L) was utilized. In the meantime, with raising the sweeping gas flow rate (from 120 to 300 L/h), the distillate was nearly 129% higher. Based on Taguchi design, the maximum permeate flux (17.3 and 17 kg/m^2^·h) was secured at 35 g/L, 0.4 L/min, 65 °C and 300 L/h, for both commercial and prepared membranes, respectively.

## 1. Introduction

Water is reckoned as the backbone for sustaining the evolution process of modernistic societies and economy. It is critical for the sake of confrontation the swift outgrowth in industrialization and human activities. The estimation of the population statistics pointed out that about 50% of the world’s population exists within a range of 100 km of an ocean. This coupling with the ongoing water scarcity issues makes oceans and seawater a virtually infinite water resource [1]. Researches manifested that about 300-million people across the world rely solely on water desalination plants for their necessities. In this context, about 18,450 desalination plants are available over 150 countries with an entire capacity capable of producing 86.8 million cubic meters/day. The most commonly employed technique for the seawater desalination is the reverse osmosis (RO). Around 60% of the entirety installed desalination plants are RO type [2]. By reflecting on the progress witnessed over the past decade, we are able to uncover recurring patterns and primary strides in the field of desalination. A way of improving the membrane system desalination is by developing an energy-efficient desalination process. As with the need for power and plant optimization, membrane distillation (MD) technique is likely to become industrially popular over the years based on the promising opportunities and prospects offered by the technique to tackle complex wastewater streams and highly saline water. However, optimizing operating parameters of the MD process is crucial for achieving consistent and reliable MD performance.

MD has well-affirmed its status as an advanced separation technology enticed the flourishing desalination research community. Substantially, separation by MD is a thermally driven process where vapour-pressure differences, engendered by the temperature variation through the membrane, is considered as the driving force for transport mechanism. The utilized membranes in the MD process are microporous in essence and have hydrophobic characteristics. A greater hydrophobic nature of the membrane could bestow a higher capability to restrain the aqueous solution penetration inside the pores, creating an interface of vapour–liquid at the pore entrance, indeed [3]. Based on Scopus scientific platform, a total of about 2900 articles were published over a span of ten years. Among this publication size on MD types, SGMD was the least investigated configuration where only forty-eight papers were dealing with SGMD. Interestingly, the majority (75%) are concentrated within the past five years. In the SGMD system, the hot solution is brought into contact with one surface of the hydrophobic porous membrane. Only vapour molecules are allowed to travel through the membrane into the permeate side where a certain inert gas is employed to sweep the vapour and condense outside of the membrane module [4]. Virtually, SGMD integrates the characteristics of both the diminished direct contact membrane distillation (DCMD) process mass-transfer resistance with the air gap membrane distillation (AGMD) low conductive heat-loss. In contrast to AGMD, the gas barrier here is sweeping the membrane (not stationary), leading to a higher permeate flux and mass transfer coefficient than that of AGMD. Aside from, the SGMD configuration showcases higher evaporation efficiency and permeate flux than the DCMD process [5].

Alongside with the merits of SGMD, and MD in general, several limitations still necessitates a fundamental understanding and adequate optimization. For instance, but not limited to; the engineering of MD membranes and modules, energetic and maintenance cost analysis, MD membrane fouling, some aspects in mass transfer models and theoretical heat [5]. In this context, operating parameters like salinity, feed temperature and the mass transfer coefficient of the membrane are predominantly defined by the membrane material characteristics, for instance, membrane’s tortuosity, porosity and thickness. Many works of literature have correlated the preparation parameters to the MD distillate flux [6,7]. This relationship is deeply discussed as elucidated in Table S1 in Supplementary Materials. However, through the preparation of membranes by phase inversion, the outright membrane characteristics are controlled by the adjustment of many major and minor variables. The prepared membrane selectivity, permeability, mechanical strength and morphology are affected by the interplay of all these parameters [8].

In this context, design of experiment (DOE) can be harnessed to impart a robust tool of statistics for the process designing and product formulation. DOE is capable to quantitatively distinguish the convenient input variable to meet a high-quality product. At the least experiments number, the DOE can be employed for optimizing the critical parameters that can endow an elaborative vision, through a minimal number of runs, over disparate variables combinations. Taguchi method relies on the orthogonal array (OA) experiments that bestow the lowest variance for the experiment with control variables having optimum settings. The technique is a statistically structured design to assess the combination of the leading factors for yielding a product. Taguchi design was harnessed to explore how unlike parameters influence the variance and mean system performance with system design optimization. Therefore, merging the optimized control variables along with DOE to recognize BEST outcomes can be acquired through the Taguchi method. OA endow a group of minimal experiments and signal-to-noise (S/N) ratios. These are log functions of the desired product and behave as objective functions for the optimization process, which is beneficial in the data analysis and the process optimum conditions calculations [9]. Herein, an attempt was made to take a meticulous glance on the role of various operating variables concerning seawater desalination by SGMD technique. Four parameters; feed temperatures, feed and sweeping gas flowrate, and feed concentration, were examined. The level of significance of every parameter on the performance of a PVDF-*co*-HFP hand-made and commercial membranes was determined. Along with that, the approach of design of experiment was implemented for optimizing the research process. Taguchi method has been harnessed to plan experiments in engineering analysis aiming to acquire data in a planned way to gain information about a given process behavior. The utmost benefit of this technique is through effort saving when saving experimental time, conducting experiments, discovering significant factors readily and diminution the cost [10]. Moreover, in the current work, it was tried to compare fabricated PVDF-co-HFP hydrophobic membrane with a common commercial hydrophobic polytetrafluoroethylene (PTFE) membrane having almost similar surface characteristics (in terms of average pore size, porosity, thickness and roughness). This comparison was conducted to evaluate the permeation and retention characteristics (performance) to see whether this membrane is worthy for the MD applications.

## 2. Experiment

### 2.1. Materials

Poly (vinylidene fluoride-co-hexafluoropropylene), having an average Mwt of 120,000, has been harnessed for membrane fabrication. PVDF-co-HFP was chosen due to its excellent thermal stability, chemical and abrasion resistance along with retaining good properties against ageing. Commercial PTFE membrane was utilized as a reference, Polyethylene glycol (PEG) with 600 Da was used as a pore former while N, N-Dimethyl acetamide (DMAC) as a solvent. All chemicals were purchased from Sigma–Aldrich, Germany.

### 2.2. Membranes Fabrication

The flat-sheet PVDF-*co*-HFP membranes were fabricated at four different host polymer concentrations via the non-induced phase separation (NIPS) method. A fixed amount (22 wt.%) of PVDF-*co*-HFP was dissolved in DMAC using a round bottom flask. The solution was mechanically stirred overnight at 40 °C until a homogenous solution was achieved. The dope solution was then degassed for five hours to eliminate air bubbles. For solution casting, a certain amount of the solution was poured onto a substrate and cas via an automated casting knife with 150 μm clearance gap. The obtained film was placed in a tap water bath for the precipitation. The membranes were then washed several times with warm deionized (DI) water (45 °C) to eliminate the residual solvent and finally stored wet at 4 °C until further use [11,12,13].

### 2.3. Membrane Characterization

Herein in this section, a comprehensive characterization process has been performed through a wide range of techniques. Scanning electron microscopy (SEM) instrument, (Zeiss EVO MA10 SEM, Carl Zeiss Promenade, Jena, Germany), was employed to observe the surface and cross-section morphology of the membranes. Prior to cross-section imaging, the samples were fractured in liquid nitrogen whereas all membranes were sputter-coated with 60% gold and 40% palladium. To analyze the surface topography; surface roughness parameters and pore size distribution, atomic force microscopy (AFM), (model AA3000, Angstrom Advanced Inc., Braintree, Boston, MA, USA), was utilized. Sessile drop method using contact angle device (CAM 110-O4W, Tainan, Taiwan) was adopted to evaluate the water contact angle measurements. At least three samples for each membrane were tested and results were averaged. The membranes porosity (ε_m_%) was determined according to the Equation (1) below [14]:

(1) $$\varepsilon_{m}\;(\%)\;=\;[1\;-\;\frac{\rho_{membrane}}{\rho_{\mathrm{PVDF}–Co\text{-}\mathrm{HFP}}}]$$

where ρ*_membrane_* and ρ _PVDF–*co*-HFP_ are the membrane and the polymer densities, respectively. The mass and volume ratios were employed to calculate the membrane density, as shown in Equation (2).

(2) $$\rho_{membrane}=\frac{m}{l\times w\times \;\delta }$$

where *m* is the mass of the membrane, l is the length of membrane, w is the width of membrane and *δ* is the thickness. The density of PVDF-*co*-HFP was taken as 1.78 g/cm^3^ as reported by the manufacturer.

Liquid Entry Pressure (LEP) of membranes was calculated using the Laplace (Cantor), Equation (3) below:

(3) $$\mathrm{LEP}\;=\;P_{\mathrm{liquid}}\;-\;P_{\mathrm{vapor}}\;=\;\frac{-2B\;\gamma^{L}\;\cos\theta }{r_{\max}\;}$$

where P_liquid_ is the pressures at the liquid side and P_vapor_ is the pressures at the vapour side, of the liquid-vapour interface at the pore entrance, θ is the contact angle between the membrane surface and the solution, γ^L^ is the surface tension of the solution, r_max_ is the largest pore size (radius). The geometric factor has referred to the coefficient B [15].

### 2.4. SGMD Experiments

**Figure 1:**
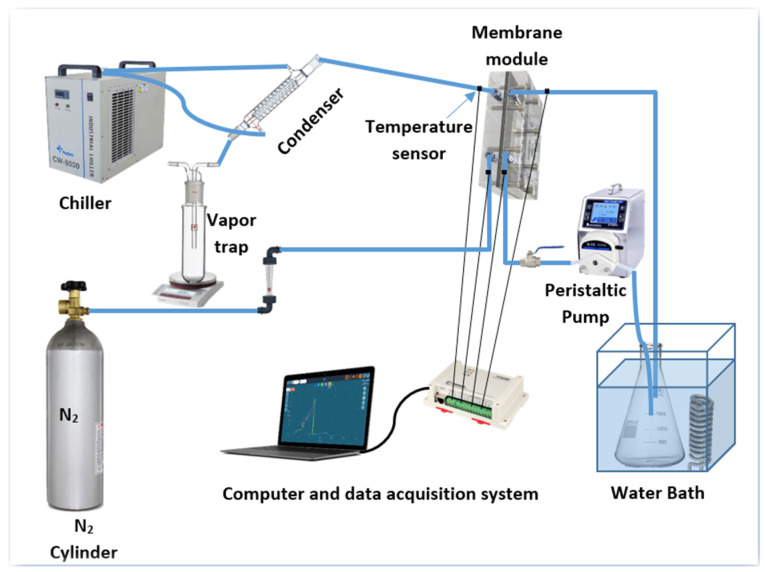
Schematic diagram of the experimental rig for the SGMD process.

The hand-made and commercial membranes were tested in a custom-made SGMD unit (Figure 1). The flat sheet membrane module was constructed of Perspex (Poly-methyl methacrylate) and comprised four closets; two for the feed (output and input) whereas the other two for the sweeping gas (output and input). Perspex was chosen to avoid the corrosive NaCl solution along with its excellent resistance to heat transfer. A more detailed description is given somewhere else [9,16]. Prior to performing the tests, the membranes were placed inside the module between the feed and permeate chambers. The hot feed solution was circulated through the feed channel side of the membrane module by a peristaltic pump (Longer pump, model BT300-1J, Longer Precision Pump Co., Ltd, Hebei, China) while the sweeping gas was passed through the permeate side. Herein, nitrogen (N_2_) was employed as sweeping gas to sweep and carry the water vapour to a condenser outside the module. The inlet and outlet module ports temperatures were continuously monitored via two sensors, attached to a multi-channel data acquisition system, type (Interface-PCI, 1712 condition, 6LM 35 sensors, Taiwan Pulse Motion, Taichung City, Taiwan). The feed hydrostatic pressure was manipulated through a control valve to prevent exceeding the LEP and to avert the membrane pores wetting. The permeate flux was estimated as given in the below equation:

(4) $$J\;=\;\frac{V\times \rho }{A\times t}$$

where J represents the permeate flux in (kg/m^2^·h), V is the freshwater volume (L), *ρ* is the water density (kg/L), t is the operational time (hour), and A is the area of the membrane and can be determined through the expression below:

A = W × L(5)

where W is the effective width of the membrane (m) and L is the effective length of the membrane (m).

Meanwhile, the feed concentration was maintained constant in the feed tank. A conductivity meter was harnessed to detect the conductivity of both the permeate water and the feed solution. The salt rejection was then calculated by the following equation:

(6) $$R\%\;=\;\frac{C_{1}-C_{2}}{C_{1}}\times \;100$$

where R is the salt retention, $C_{1}$ is the feed concentration (mg/L), and $C_{2}$ is the concentration of the permeate (mg/L).

### 2.5. Experimental Design Using Taguchi Method

The experimental design approach can be defined as the techniques devoted for directing the experiments choice to be executed in an efficacious way [17]. Taguchi method has broadly established its way as a method for the design of experiments. It has well-demonstrated reliability for achieving high-quality products at minimal cost indeed. However, the adoption of an adequate orthogonal array (OA) reckons on the process parameters degrees of freedom (DOF), depending on the number of levels of versatile parameters [18,19]. Herein, since there are four parameters where each is at three levels, DOF can be calculated as below:

DOF = P × (L_9_ − 1) (7)

where P is the parameters number and L_9_ is the levels number.

DOF = 4 × (3 − 1) = 8

Basically, the DOF of the OA must be higher than or, at minimal, an equal to the parameters of the process. Four parameters alongside three-level columns with 8 DOF are what the standard L_9_(3^4^) orthogonal array inclose. Therefore, the orthogonal array employed in the current study has four columns and nine rows. Additionally, three operating parameters, each one at three levels, were adopted to estimate the water vapour flux. The chosen factors for the study are listed in Table 1. According to Taguchi technique requirements, nine experiments have been performed at disparate parameters, i.e., the nine rows correspond to the number of the tests, with four columns represent the investigated parameters at three levels for each one, as given in Table 2. The statistical methodology ANalysis Of VAriance (ANOVA) will be utilized to analyze the experimental data and to estimate the contribution of each factor on the entire membranes’ performance. ANOVA is an omnibus statistical test (collection of statistical models) to provide a way to find out if the experimental results are significant, according to the law of total variance (the expectation of the squared deviation of a random variable from its mean), where the noticed variance in a specific variable has divided into components assigned to various variation sources. In other words, it helps to figure out statistically, to assess the participation of each factor (operating conditions) to the obtained output (permeate flux).

## 3. Results and Discussion

### 3.1. Membranes Characteristics

**Figure 2:**
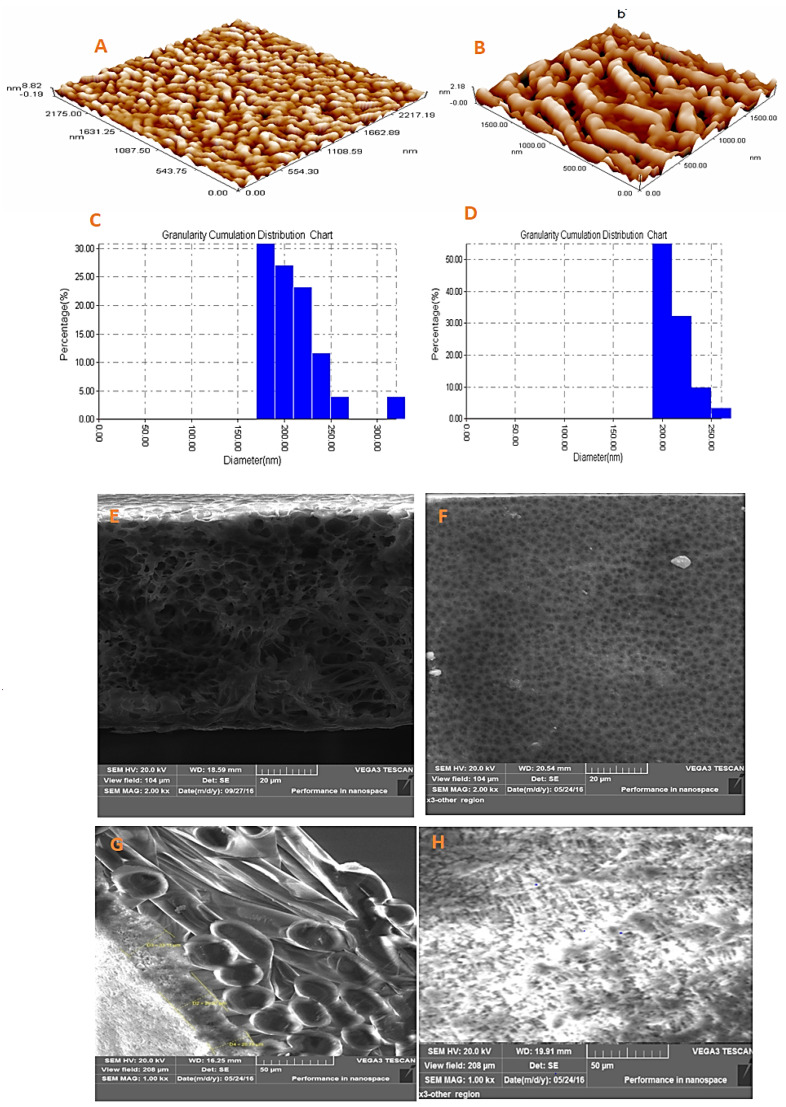
Surface topography (Top) and pore size distribution (Bottom) images determined by AFM for fabricated PVDF-*co*-HFP (**A**,**C**) and commercial PTFE (**B**,**D**) and (**E**) and (**F**) are the cross-sections and top surface SEM images of the PVDF-g-HFP membranes while (**G**) and (**H**) are the cross-sections and top surface SEM images of the commercial membranes.

Surface characteristics including roughness and pore size are pivotal characteristics for determining any membrane’s performance since they have a crucial impress on the liquid mass transfer. Herein, each PVDF-*co*-HFP and PTFE membrane have been undergone to an extensive surface analysis utilizing AFM by contact mode with an appropriate silicone tip. Measurements comprised an evaluation of the membrane’s topography and the lateral force which is the friction forces among the tip and surface that give rise to the cantilever’s torsion. This torsion magnitude has been reflected by the left-right signal of the photodetector. Also, the deflection where the cantilever flexes due to the fall and rise of the sample topography. The magnitude of obtained deflection was reflected by the up-down signal of the photodetector. Surface topography for both fabricated and commercial membranes was depicted in Figure 2A,B, respectively. The IMAGER 4.31 software (Angstrom Advanced Inc., Stoughton, MA, USA) was employed to obtain statistics about the pore size distribution for each sample surface. As revealed by the software, the PVDF-*co*-HFP membrane manifested an average pore size of 0.20 micron, a slightly smaller than that of commercial PTFE (0.22) micron. The granularity cumulation distribution of the pores was illustrated in Figure 2C,D, respectively. Other membrane characteristics (Root average arithmetic roughness (Ra), thickness, porosity, LEP, and hydrophobicity) are tabulated in Table 3 below. Whereas, the top surface and the cross-sectional imaging by the spectroscopy were shown in Figure 2. Figure 2E,F are the cross-sectional and upper surface SEM images of the PVDF-g-HFP membranes while Figure 2G,H are the SEM images of the commercial membranes.

### 3.2. Role of Operating Parametric on SGMD

Regardless of membrane characteristics, the efficiency of the SGMD process can considerably be influenced by the operating conditions. In order to evaluate the performance of SGMD, a set of experiments were performed and the impacts of the operational parameters on the membrane’s permeation characteristics were investigated. In the current work, four parameters, at three different levels, were taken into account such as feed flow rate (0.2–0.6 L/min), feed temperature (45–65 °C), sweeping gas flow rate (120 to 300 L/h) and feed concentration (35–100 g/L). It should be noted here that the fabricated membrane with 22 wt.% PVDF-co-HFP was adopted after a series of trial and error (not presented in the current work). This was conducted aiming to produce a membrane with characteristics close to that of the commercial PTFE membrane. Indeed, that would facilitate the performance simulation as illustrated in the subsequent sections.

#### 3.2.1. Influence of Feed Temperature

**Figure 3:**
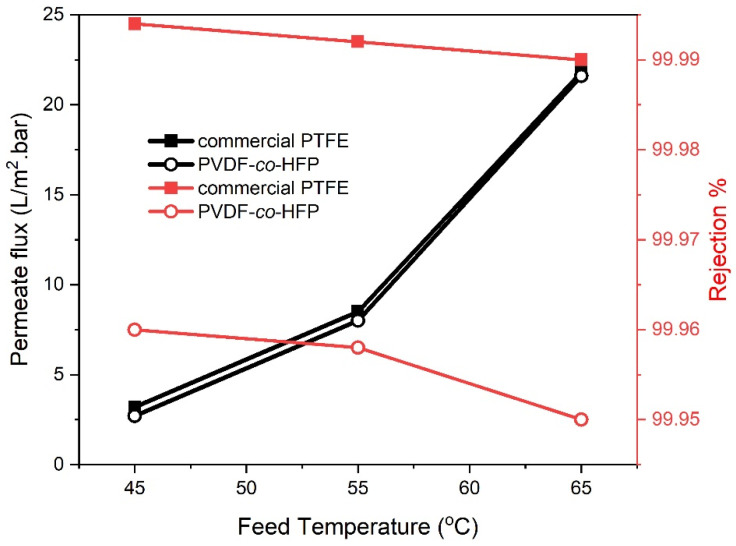
Effect of feed temperature on permeate flux and salt rejection for 35 g/L NaCl feed solution, 0.6 L/min feed flow rate and 300 L/h sweeping gas flow rate for PTFE and PVDF-co-HFP membranes.

The variation of distillate flux with feed temperatures was illustrated in Figure 3, for the two proposed hydrophobic PTFE and PVDF-*co*-HFP membranes. The feed temperature adopted here was ranged from (45 to 65 °C) while feed flow rate, feed concentration and sweeping gas flow rate were maintained constant at 35 g/L, 0.6 L/min and 300 L/h, respectively. As can be identified, the distillate flux rate raised by the feed temperature. This is a common behaviour for all MD systems and repeatedly been elucidated based on Antoine’s equation where the higher temperature could bestow an exponential increase in the saturated vapour pressure of volatile molecules. This would indeed translate higher operating temperature into a straightforward driving force for MD processes to endow an exponentially increased permeate flux [20]. These results have well agreed with the other literature reported by [21]. For both membranes, the distillate flux was almost identical and increased by about (~265)% with raising the feed temperature from 45 to 55 °C while was nearly (~680)% when the feed temperature raised from 45 to 65 °C. Meanwhile, comparing the retention characteristics of the prepared and commercial membrane, at 65 °C, revealed a rejection value of 99.95% and 99.99% with a conductivity of 23 and 5 μs/cm, respectively. These retention values were slightly lower if compared with those been manifested at 45 °C in contra to what has been witnessed for the conductivity. This suggested that the temperatures have a slight impact on the pore wetting of the membranes.

#### 3.2.2. Effect of Feed Flow Rate

**Figure 4:**
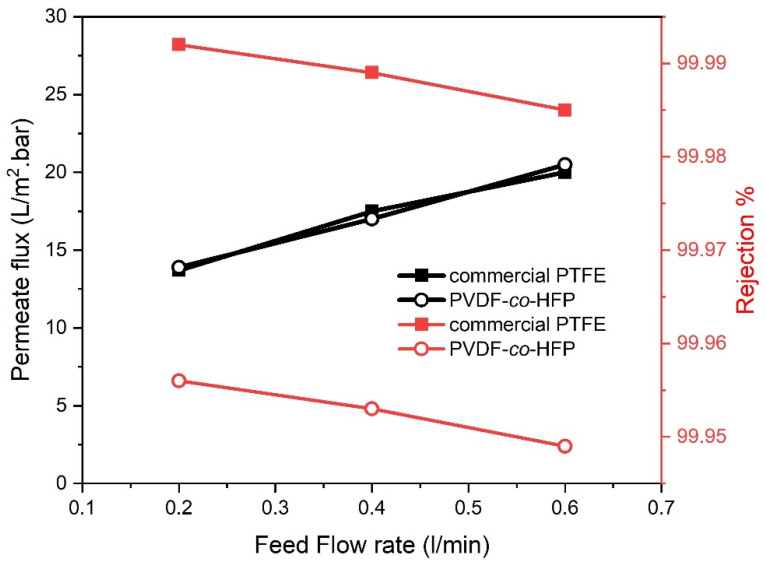
Effect of feed flowrate on permeate flux and salt rejection for 35 g/L NaCl feed solution, 65 °C feed temperature and 300 L/h sweeping gas flow rate for PTFE and PVDF-co-HFP membranes.

**Figure 5:**
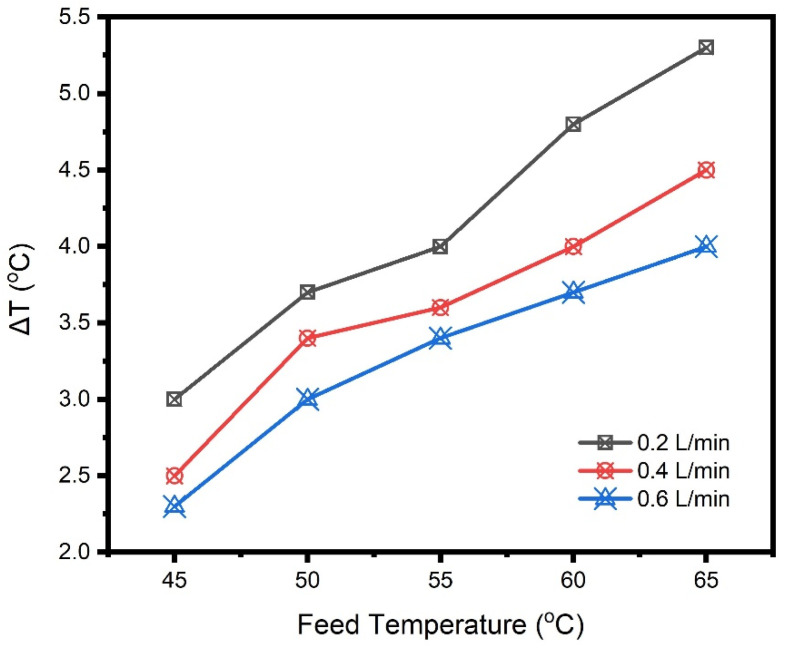
Variance between inlet and outlet feed temperatures versus the inlet feed temperatures for 35 g/L NaCl solution and 300 L/h sweep gas flow rate.

Virtually, the feed flow rate is a decisive operating variable that inspires MD performance. To determine the impact of flow rate on the SGMD unit, a series of tests were performed through varying the feed flow rate (0.2 to 0.6 L/min) at 35 g/L NaCl solution, 65 °C feed temperature and 300 L/h sweep gas flow rate. Like trends observed in previous literature [16,22,23], the distillate flux increased almost linearly with rising the flow rate, as disclosed below in Figure 4. As the flow rate increases from 0.2 to 0.4 and 0.6 L/min, about 27 to 46% and 22 to 47.5% increment has been bestowed on water permeability characteristics of commercial PTFE and fabricated PVDF-*co*-HFP membranes, respectively. This rise in the flux values was ascribed to the Reynolds number accretion. This indeed causes an enhanced flow mixing inside the channels and mass transfer as a consequence of turbulence. In turn, this flow could diminish both concentration and temperature boundary layers thickness, giving rise to a higher flux due to the downgraded boundary layer resistance [5,24]. In this context, the dissimilarities, in the recorded temperatures between the hot feed inlet and outlet (ΔT) against the feed temperature, are displayed in Figure 5. A higher ΔT was observed at the lower feed flow rate (0.2 L/min) as a result of protracted residence of the feed solution when passed through the membrane module and vice versa. Hence, 0.6 L/min flow rate have revealed the shorter residence inside the module, and in turn, having a greater feed outlet temperature to promote Reynold’s number and the driving force.

Back to Figure 4, the impact of increasing the flow rate has indicated that salt retention was slightly affected, for both membranes. This could be ascribed to the pressure difference at the different flow rates where the high flow rate may have allowed a slight wetting to occur under the fluctuated pressures [25]. Therefore, lower rejection values, at a higher flow rate, were observed during the SGMD processes. Comparing the retention results, increasing the feed flow rate value, from 0.2 L/min to 0.6 L/min, have resulted in 99.956% and 99.949% rejection by the fabricated membrane while 99.992% and 99.985% were achieved through the commercial PTFE membrane.

#### 3.2.3. Influent of Feed Concentration

**Figure 6:**
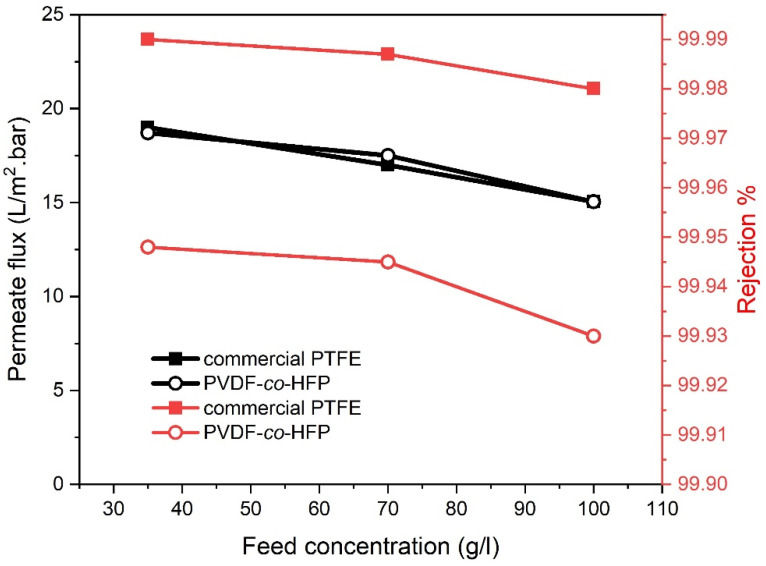
Effect of feed concentration on permeate flux and salt rejection at 65 °C feed temperature, 0.6 feed flowrate and 300 L/h sweep gas flow rate for PTFE and PVDF-co-HFP membranes.

Similar to other membrane separation processes, all MD configurations are oversensitive to the feed concentration [26]. Unlike feed concentrations (35, 70 and 100 g/L) at 0.6 L/min feed flow rate, 300 L/h sweeping gas flow rate and 65 °C feed temperature were harnessed during the experiments. As depicted in Figure 6 below, the results manifested that distillate flux decreases upon increased concentrations. About 18.7% permeate flux reduction, for the PVDF-*co*-HFP membrane, was recorded when rejecting 35 g/L NaCl feed solution. Further straightforward flux decline by 24% and 34.5% was witnessed when higher saline feed concentrations (70 and 100 g/L respectively) are employed. Close to what has been spotted when utilizing fabricated membranes, an experimental test carried through commercial PTFE membrane has revealed a flux decline of 17.4%, 26% and 34.4% for the saline feed concentrations of 35, 70 and 100 g/L, respectively. This was assigned to the role of concentration polarization as an additional boundary layer has been formed. This concentration boundary layer alongside temperature boundary layer performs as further resistance to the vapour molecules transfer and diminish the evaporation driving force, indeed [7,27,28,29].

In the meantime, the permeate conductivity has been increased sharply reaching 35 and 17 μs/cm for the commercial and prepared membranes, respectively. Thus, the salt rejection was lowered at higher NaCl concentration. The augmentation in the conductivity of the permeate was related to the diminished LEP at the higher concentrations. Water could only penetrate the membrane at a pressure overtakes the membrane LEP. However, at 100 g/L feed concentration, the salt rejection values are still higher than 99.98% and 99.93% for commercial and fabricated membranes, respectively.

#### 3.2.4. Influence of the Sweeping Gas Flow Rate

**Figure 7:**
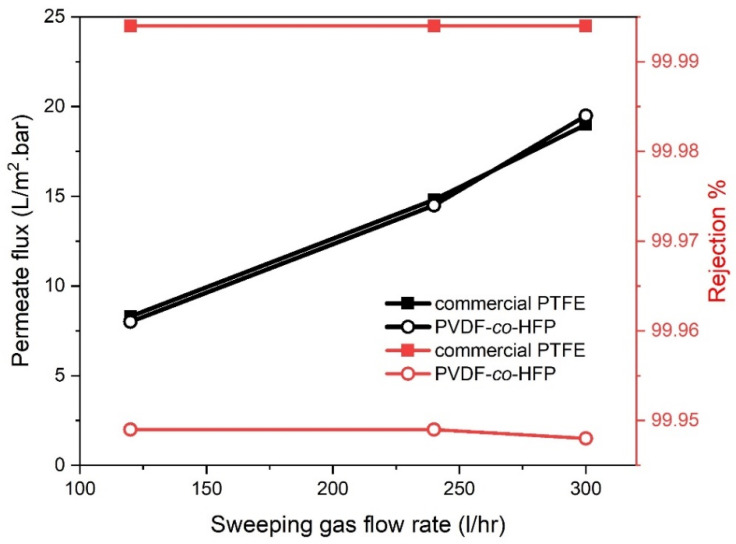
Effect of sweeping gas flow rate on permeate flux and salt rejection at 65 °C feed temperature, 0.6 feed flowrate and 35 g/L feed concentration for PTFE and PVDF-co-HFP membranes.

In present SGMD configuration, nitrogen as an inert gas was utilized to sweep the vapour from the permeate side of the membrane whereas condensation occurred out the membrane at ambient conditions. Therefore, the sweeping gas flow rate has potentially controlled the MD process. A slight difference in the sweeping gas flow rate imparts considerable impacts on the permeate flux [30]. Figure 7 displayed how can the sweeping gas influence the distillate flux in an SGMD unit. Herein, the sweeping gas flow rate was ranged from 120 to 300 (L/h) while the feed flow rate and the NaCl concentrations were fixed at 0.6 L/min and 35 g/L, respectively. As demonstrated by the results, permeate flux has a proportional correlation with the sweeping gas flow rate. For the commercial membrane, the permeate flux was raised by about 78% with increasing the sweeping gas flow rate from 120 to 240 L/h while the distillate was nearly 129% at 300 L/h. whilst almost identical permeate flux values were obtained from PVDF-co-HFP membranes, under the identical operational conditions. This augmentation in the permeate flux versus the sweeping gas flow rate could be elucidated by the declined role of the temperature polarization effect. The temperature polarization is basically counting on the fluids dynamic characteristics contiguous to the membrane and found the permeate side within the SGMD module. As the sweeping gas flow rate rises, the Reynolds number increases. The temperature at the surface of the membrane and in the bulk permeate are nearly the same. Ultimately, the resistance of the thermal boundary layer is reduced, and the heat transfer increases with minimized temperature polarization effects, leading to higher permeate flux values. It is worth mentioning that literature has reported an increase in the temperature polarization coefficient with increasing air velocity, and hence, indicating a decrease in the temperature polarization effect [31]. It can be noted that the permeate produced from prepared membrane and commercial had a conductivity of about 19.8 and 5 μs/cm, respectively. This indeed has resulted in high retention through them by 99.94% and 99.99% respectively, as depicted in Figure 7.

### 3.3. Data Analysis through Taguchi Design

Taguchi method is a robust statistical design method founded to enhance the quality of manufactured goods. It has been introduced for the designing of experiments to probe how unlike parameters influence the variance and mean of any process performance characteristic that determines how good the process is functioning. The technique comprises minimizing the variation in a process via the robust design of experiments (DOEs) [32]. The robust design objective is to enhance the quality of the product by reducing the impacts of variation. This was conducted without eliminating causes which are either quite expensive or difficult to dominate. The end result here is to obtain a design with minimal sensitivity to the variations in the unrestrainable factors. The main philosophy of the Taguchi method is; quality must be designed into the product and not inspected out of it. Preferably, quality is carried out by reducing the target’s deviation, and the quality cost should be detected as a function of deviation from the targeted system.

It is well known that the DOE relied on how various design factors influence mean results, but Taguchi’s DOE focuses on the variance rather than the mean. Moreover, the noise is treated by the former as an extraneous factor, while it is considered as a central point of its analysis by the latter. According to the Taguchi design, an L_9_(3^4^) orthogonal array (four variables in three levels, Table 1) was examined. The results of the experiments are shown in Table 4 and Table 5 for both commercial and prepared membrane, respectively. The flux was measured after nearly 180 min. The better the performance characteristics, the greater the permeate flux.

Since the experimental design is orthogonal, the role of each operating parameter on the major effect (permeate flux) at different levels could be separated. Table 4 and Table 5 manifested the response value for each level for both commercial and prepared membranes, respectively. It can be seen that the maximum permeate flux at 35 g/L, 0.4 L/min, 65 ℃ and 300 L/h are 17.3 and 17 (kg/m^2^·h) for both commercial and prepared membranes, respectively which represent the best-operating conditions for these experiments (better performance characteristic).

### 3.4. Analysis of Data of Commercial Membrane

Taguchi design harnesses the ratios of signal-to-noise (S/N) as the response variables, that makes a trade-off amongst setting the mean to an eligible level while maintaining the variance low. The S/N ratio elucidated under disparate situations as “Target is Best”, “Smaller is Better”, and “Larger is Better”. For a Larger is a Better situation, the S/N ratio set as:

(8) $$S/N=-\;10\;log_{10}[\sum {\left(\frac{1}{y_{i}^{2}}\right)}^{2}\frac{1}{n}\;]$$

where *y_i_*: response at each observation while *n*: number of observations. The target of the noise factors is to induce the system performance to depart from its objective value. In the current work, three operational variables, each one at three levels, were chosen to assess the flux. The required factors to be examined are given in Table 2.

According to the Taguchi technique principles, nine experiments have been carried out regarding different parameters. According to that, the nine rows coincided to the number of the experiments while the four columns denoted the studied parameters, at three levels for each parameter, as given in Table 6. After conducting the experiments based on the Taguchi method, the results were converted into S/N ratio values. The final L_9_(3^4^)-OA displaying response values and their corresponding S/N ratio values for water vapour flux are shown in Table 6. Taguchi method and analysis of variance “ANOVA” were applied to analyze the experimental data and to define the participation of every factor on the membrane performance. The results of experiments and the ratio of signal to noise (S/N) are illustrated in Table 6. As could be seen, the maximum flux was 17.3 kg/m^2^·h at 35 g/L, 0.4 L/min, 65 ℃ and 300 L/h which represented the optimum operating conditions. Krishankant and co-workers [33] reported that regardless of the category of the performance characteristics and based on Equation (6), a greater S/N value corresponds to the best operating conditions. As could be noticed in Table 7, the greater ratio (S/N) was 24.761, at the same operating conditions mentioned above.

**Figure 8:**
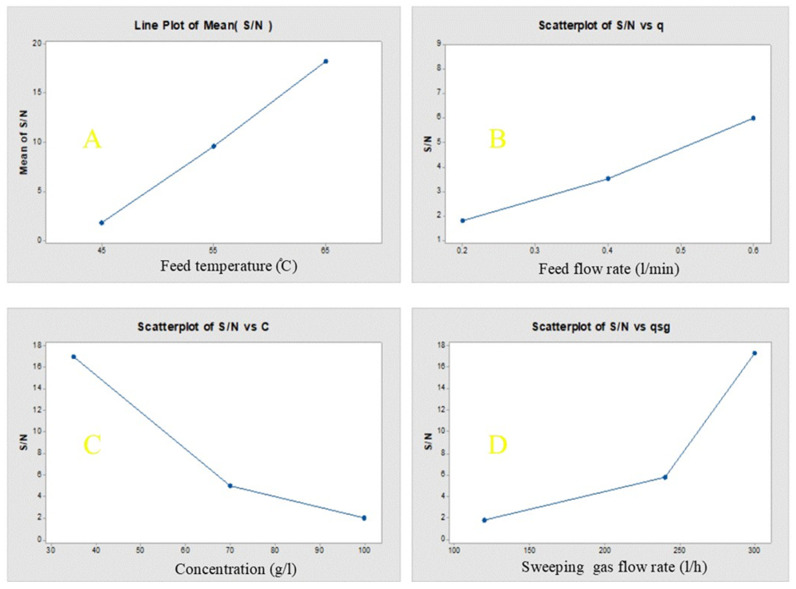
Effect of operating variables on S/N ratio: (**a**) Feed temperature; (**b**) Feed flow rate; (**c**) Concentration; (**d**) Sweeping gas flow rate.

The influence of each parameter on the permeate flux in the SGMD process, as given by Minitab 17 software, was shown in Figure 8. It represented the main effect plots, based on the average values of each experimental run for system permeation flux. The reasonable trend of permeate flux, concerning the corresponding operating conditions, has indicated that the permeate flux increased with increasing feed temperature, feed flow rate and sweep gas flow rate (Figure 8A,B,D). In the meantime, it decreased with increasing feed concentration as shown in Figure 8C. The figure has also represented the contribution of each parameter of operating conditions on the permeate flux. It can be concluded that the feed temperature was the most significant factor, while the feed flow rate showcased the lowest influence on the permeate flux. Based on the levels of operational parameters as shown in Table 1, and their experimental results are given in Table 6, Minitab 17 software was used for statistical analysis. Table 7 represented the ANOVA results, comprising of the degree of freedom (DOF), according to Equation (7) the sum of squares (SS), (is the sum of squares of deviation from the mean of all parameter values), mean square (variance) based on the variation among the sample means (corresponding to the model) and the variation within the samples (corresponding to the error), factor variance to error (F) and contribution of each factor to the response (P). The table manifested that the maximum F-value and P are related to temperature, concluding that this parameter has the maximum influence on the permeate flux.

Table 8 shows the mean flux and the S/N ratio at all levels of factors as given by Minitab 17 software and shows the effect of each factor on flux and S/N ratio. It can be seen from Table 8 that the best performance of SGMD process based on the analysis of S/N ratio was obtained at 65 °C and corresponds to the permeation flux of 20.3299 kg/m^2^·h. It can be shown from Table 8 that the S/N ratio increases with an increase in feed temperature, so the optimum feed temperature is level 3 (65 °C). This ratio also increases with increase in sweep gas flow rate so the optimum sweep gas flow rate is level 3 (300 L/h) and it increases with the increase of flow rate so the flow rate is level 3 (0.6 L/min) and it decreases with increasing concentration so the optimum concentration is level 1 (35 g/L).

### 3.5. Predicted Model

**Figure 9:**
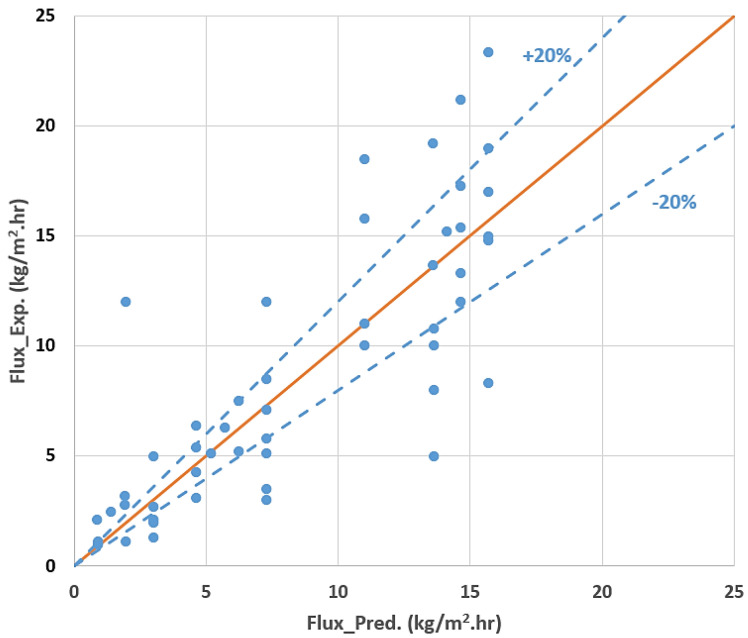
Predicted versus experimental values of permeat flux.

A Minitab-17 regression model for prediction of permeation flux was created by function nonlinear curve fitting. The permeate flux was modelled as the dependent variable while the feed temperature, feed flow rate, sweep gas flow rate and feed concentration were considered as the independent variables. The Minitab predicted model for the present SGMD system based on all experimental data gives the following relation:

J = 33.5 − 1.53T + 1.72q − 0.02204q_sg_ + 0.0589C + 0.0182T^2^ (9)

where J is the permeate flux represented the dependent variable T, q_sg_, q, C is the feed temperature in °C, sweep gas flow rate (L/h), feed flow rate in L/min and feed concentration g/L, respectively, and represented the independent variables. The predicted and measured permeate fluxes were given in Figure 9.

The proposed model from the Taguchi technique has the coefficient of correlation (R-Square) of 0.67, meaning that 67% of the variation in permeate flux is captured by variation in feed temperature, feed flow rate, sweep gas flow rate and feed concentration. This implies that the variation in permeate flux was explained by variation in feed temperature, feed flow rate, sweep gas flow rate and feed concentration, taking into account the experimental data size and the number of independent variables.

## 4. Conclusions

Experimental and theoretical work on SGMD system has been conducted to speculate and optimize its performance for seawater desalination. The influence of four pertinent operational variables, at three different levels, were taken into account such as feed temperature(45–65 °C), feed flow rate (0.2–0.6 L/min), feed concentration (35–100 g/L), and sweeping gas flow rate (120 to 300 L/h). A hand-made casted PVDF-*co*-HFP membrane was successfully fabricated via the classical NIPS technique and employed for the current work along with a commercial PTFE membrane. Both membranes were fully characterized in terms of their surface and permeation characteristics including the roughness, surface and cross-section imaging, LEP, thickness, porosity, pore size and pore size distribution. The fabricated membrane manifested 0.2 microns, 1.3, 78% and 98 as pore size, Ra, porosity and contact angle values, respectively.

The theoretical modelling and optimization of the SGMD system were performed based on the DOE and the Taguchi technique by an orthogonal array (OA). Taguchi method and analysis of variance “ANOVA” were applied to analyze the experimental data and to determine the contribution of each factor on the membrane performance. From the contribution of each operating conditions parameters on the permeate flux, it can be concluded that the feed temperature most the most significant factor, while the feed flow rate showcased the lowest influence on the permeate flux. Virtually, the maximum permeate flux was obtained at 35 g/L, 0.4 L/min, 65 ℃ and 300 L/h. The distillate values were 17.3 and 17 kg/m^2^·h for both commercial and prepared membranes, respectively. These conditions represented the best-operating conditions during the experiments (better performance characteristic). Besides that, a Minitab-17 regression model for prediction of permeation flux was created by function nonlinear curve fitting. The proposed model from the Taguchi technique has a correlation coefficient (R-Square) of 0.67, meaning that 67% of the variation in permeate flux was captured by variation in feed temperature, feed flow rate, sweep gas flow rate and feed concentration.

## Figures and Tables

**Table 1:** Factors and their levels in the experimental design.

Level	Feed Temperature (°C)	Feed Concentration (g/L)	Sweep Gas Flow Rate (L/h)	Feed Flow Rate (L/min)
1	45	35	120	0.2
2	55	70	240	0.4
3	65	100	300	0.6

**Table 2:** Taguchi L_9_ orthogonal array.

Run	Operating Parameters			
	C (g/L)	T (°C)	q (L/min)	q_Sg_ (L/h)
1	35	45	0.2	120
2	70	45	0.4	240
3	100	45	0.6	300
4	70	55	0.2	300
5	100	55	0.4	120
6	35	55	0.6	240
7	100	65	0.2	240
8	35	65	0.4	300
9	70	65	0.6	120

C: feed concentration, T: feed temperature, q: feed flow rate, q_sg_: sweep gas flow rate.

**Table 3:** Characteristics of the membranes.

Characteristics	PVDF-*co*-HFP	Commercial
Average pore size (μm)	0.202	0.22
Mean roughness (Ra) (nm)	1.3	1.27
Thickness (μm)	96	96
Porosity Ɛ %	0.78	0.78
LEP (kPa)	87	
Contact angle	98	114

**Table 4:** Taguchi L_9_(3^4^) OA, and result of experiments for commercial membrane.

Run	Operating Parameters				Flux (kg/m_2_·h)	Rejection %
	T (°C)	Q (L/min)	C (g/L)	q_S_ (L/hr)		
1	45	0.2	35	120	1.23	99.993
2	45	0.4	70	240	1.5	99.992
3	45	0.6	100	300	2	99.991
4	55	0.2	70	300	4.8	99.989
5	55	0.4	100	120	3	99.99
6	55	0.6	35	240	5.8	99.988
7	65	0.2	100	240	7.9	99.987
8	65	0.4	35	300	17.3	99.986
9	65	0.6	70	120	8.2	99.987

**Table 5:** Taguchi L_9_(3^4^) OA, and result of experiments for Prepared membrane.

Run	Operating Parameters				Flux (kg/m_2_·h)	Rejection %
	T (°C)	Q (L/min)	C (g/L)	q_S_ (L/hr)		
1	35	45	0.2	120	1.4	99.956
2	70	45	0.4	240	1.7	99.95
3	100	45	0.6	300	2	99.949
4	70	55	0.2	300	4.9	99.947
5	100	55	0.4	120	2.98	99.944
6	35	55	0.6	240	6	99.946
7	100	65	0.2	240	7.88	99.93
8	35	65	0.4	300	17	99.934
9	70	65	0.6	120	8.09	99.93

**Table 6:** Taguchi L_9_(3^4^) OA, and result of experiments.

Run	Operating Parameters				Flux (kg/m^2^·h)	S/N
	C (g/L)	T (°C)	Q (L/min)	q_sg_ (L/hr)		
1	35	45	0.2	120	1.23	1.798
2	70	45	0.4	240	1.5	3.522
3	100	45	0.6	300	2	6.0206
4	70	55	0.2	300	4.8	13.6248
5	100	55	0.4	120	3	9.5424
6	35	55	0.6	240	5.8	15.2686
7	100	65	0.2	240	7.9	17.9525
8	35	65	0.4	300	17.3	24.761
9	70	65	0.6	120	8.2	18.2763

**Table 7:** Parameters of the statistical analysis.

Degree of Freedom	Factor	Sum of Square	Variance	F	P
2	Concentration	142.5	97.03	4.51	11.17 %
2	Temperature	1115.6	861.17	26.71	44.53 %
2	Flow rate	46.12	39.26	2.3	3.21 %
2	Sweep gas flow rate	752.6	388.65	12.13	21.11 %
8	Error	188.7	21.81		
16	Total	2245.52			

**Table 8:** Mean flux and S/N values at all levels of operating variables obtained from the Taguchi method.

Parameters	Level	Mean Flux (kg/m^2^·h)	S/N (Larger the Better)
Temperature (°C)	45	1.5767	17.78
	55	4.5333	18.8119
	65	11.1333	20.3299
Sweep gas flow rate (L/h)	120	8.11	13.9425
	240	4.8333	15.8076
	300	4.300	16.1719
Feed flow rate (L/min)	0.2	4.643	11.1252
	0.4	7.266	12.6084
	0.6	5.333	13.1885
Concentration (g/L)	35	4.1433	9.8723

## Supplementary Materials

The following are available online at https://www.mdpi.com/2077-0375/10/10/254/s1, Table S1: Operating conditions versus permeate flux in SGMD configuration.

## Nomenclatures

ε_m_	porosity
A	membrane effective area (m^2^)
AFM	atomic force microscopy
C_1_	concentration of the feed NaCl solution (mg/L)
C_2_	concentration of permeate solution (mg/L)
FS	flat sheet
HF	hollow fiber
ID	inside diameter
J	permeate flux (kg/m^2^·h)
L	effective length (m)
LEP	liquid entry pressure
*m*	the mass of the membrane
OD	outer diameter
q	flowrate (L/min)
q_sg_	sweep gas flow rate
r	pore radius
SEM	scanning electron microscopy
Tf	feed temperature (°C)
ti	operational time in (h)
V	volume of water (L)
W	effective width (m)
*δ*	thickness
*ρ*	water density in (kg/L)
l	the length of membrane
w	the width of membrane
DOE	Design of Experiment
