# Supplementary material for: A Systematic Framework for Optimizing a Sweeping Gas Membrane Distillation (SGMD)
Source: Membranes (Basel). 2020 Sep 24;10(10):254. doi: 10.3390/membranes10100254 (PMC7598666; doi:10.3390/membranes10100254)
Supplement: Supplementary file 1 [file membranes-10-00254-s001.pdf]

A Systematic Framework for Optimizing a Sweeping Gas Membrane Distillation (SGMD)

Nawras N. Safi [1], Salah. S. Ibrahim [1], Nasser Zouli [2], Hasan Shaker Majdi [3], Qusay F. Alsalhy [1,*], Enrico Drioli [4] and Alberto Figoli [4]

[1] Membrane Technology Research Unit, Chemical Engineering Department, University of Technology, Alsinaa Street 52, Baghdad 10066, Iraq; nawras.nabeel@gmail.com (N.N.S.); salah.s.ibrahim@uotechnology.edu.iq (S.S.I.)
[2] Department of Chemical Engineering, Jazan University, P.O. Box 706, Jazan 45142, Saudi Arabia; nizouli@jazanu.edu.sa
[3] Department Of Chemical Engineering and Petroleum Industries, AlMustaqbal University College, Babylon 51001, Iraq; hasanshker1@gmail.com
[4] Institute on Membrane Technology, National Research Council (ITM-CNR), Rende (CS) 87030, Italy; e.drioli@itm.cnr.it (E.D.); a.figoli@itm.cnr.it (A.F.)
* Correspondence: 80006@uotechnology.edu.iq; Tel.: +964-7901730181

Received: 24 July 2020; Accepted: 15 August 2020; Published: date

Table S1. Operating conditions versus permeate flux in SGMD configuration.

**Ref**	**Type membrane and module**	**Characteristic of membrane**							**Operating condition**				**J (kg/m².h)**
		**ID mm**	**OD mm**	**δ μm**	**L (mm)**	**A (cm²)**	**ε(%)**	**r(μm)**	**feed**	**Tf (C)**	**q (L/min)**	**q_{sg} (L/h)**	
[1]	PTFE-HF	0.9	2	∅	240	602	62	0.8	Isopropanal and water	46	0.24	120	1.8
[2]	PP-HF	1.8	2.6	178	∅	∅	70	0.2	Pure water	65	0.4	85	0.03
[3]	PTFE-FS	∅	∅	150	∅	50	70	0.45	100 mg/l Ammonia solution	75	0.25	300	10
[4]	PTFE-FS	∅	∅	55	∅	56	69	0.198	Brine	50	0.1	95	0.15
[5]	PTFE-FS	∅	∅	100	∅	55.3	80	0.45	30 g/L NaCl solution and seawater	71.6	0.75	230	2.05

[6]	PTFE-FS	Ø	Ø	178	Ø	55.3	80	0.45	30 g/L sodium chloride	69	0.67	150	1.5
[7]	PTFE-FS	Ø	Ø	175	Ø	0.0009	Ø	0.18	NaCl aqueous solution	80	0.5	300	15
[8]	PTFE-HF	1.18	1.7	240	150	200	Ø	0.417	pure water	40	0.2	100	0.2-0.14
[9]	PVDF-HF	0.23	0.33	Ø	127	5700	55	0.1	Brine	70	0. 25	100	4.67
[10]	PVDF-CO-HFP	Ø	Ø	Ø	Ø	Ø	Ø	Ø	Prepared membrane	Ø	Ø	Ø	Ø
[11]	PVC	Ø	Ø	Ø	Ø	Ø	Ø	Ø	Prepared membrane	Ø	Ø	Ø	Ø
[12]	PVDF	Ø	Ø	Ø	Ø	Ø	Ø	Ø	Prepared membrane	Ø	Ø	Ø	Ø
[13]	PTFE	Ø	Ø	Ø	Ø	Ø	Ø	Ø	Distillate water	71	Ø	Ø	1.3

References:

[1] C.H. Lee, W.H. Hong, Effect of operating variables on the flux and selectivity in sweep gas membrane distillation for dilute aqueous isopropanol, Journal of Membrane Science, 188 (2001) 79-86.

[2] M. Khayet, P. Godino, J.I. Mengual, Theory and experiments on sweeping gas membrane distillation, Journal of Membrane Science, 165 (2000) 261-272.

[3] Z. Xie, T. Duong, M. Hoang, C. Nguyen, B. Bolto, Ammonia removal by sweep gas membrane distillation, Water research, 43 (2009) 1693-1699.

[4] K. Charfi, M. Khayet, M.J. Safi, Numerical simulation and experimental studies on heat and mass transfer using sweeping gas membrane distillation, Desalination, 259 (2010) 84-96.

[5] M. Khayet, C. Cojocaru, A. Baroudi, Modeling and optimization of sweeping gas membrane distillation, Desalination, 287 (2012) 159-166.

[6] M. Khayet, C. Cojocaru, Artificial neural network model for desalination by sweeping gas membrane distillation, Desalination, 308 (2013) 102-110.

[7] L. Wang, B. Li, X. Gao, Q. Wang, J. Lu, Y. Wang, S. Wang, Study of membrane fouling in cross-flow vacuum membrane distillation, Separation and Purification Technology, 122 (2014) 133-143.

[8] S. Shukla, N.E. Benes, I. Vankelecom, J. Méricq, M. Belleville, N. Hengl, J.S. Marcano, Sweep gas membrane distillation in a membrane contactor with metallic hollow-fibers, Journal of membrane science, 493 (2015) 167-178.

[9] V. Karanikola, A.F. Corral, H. Jiang, A.E. Sáez, W.P. Ela, R.G. Arnold, Sweeping gas membrane distillation: numerical simulation of mass and heat transfer in a hollow fiber membrane module, Journal of Membrane Science, 483 (2015) 15-24.

[10] Q.F. Alsalhy, K.T. Rashid, S.S. Ibrahim, A.H. Ghanim, B. Van der Bruggen, P. Luis, M. Zablouk, Poly (vinylidene fluoride-co-hexafluoropropylene)(PVDF-co-HFP) hollow fiber membranes prepared from PVDF-co-HFP/PEG-600Mw/DMAC solution for membrane distillation, Journal of Applied Polymer Science, 129 (2013) 3304-3313.

[11] M.A. Tooma, T.S. Najim, Q.F. Alsalhy, T. Marino, A. Criscuoli, L. Giorno, A. Figoli, Modification of polyvinyl chloride (PVC) membrane for vacuum membrane distillation (VMD) application, Desalination, 373 (2015) 58-70.

[12] W.-T. Xu, Z.-P. Zhao, M. Liu, K.-C. Chen, Morphological and hydrophobic modifications of PVDF flat membrane with silane coupling agent grafting via plasma flow for VMD of ethanol–water mixture, Journal of Membrane Science, 491 (2015) 110-120.

[13] A. Essed, Modeling of Membrane Distillation-assisted Crystallization: Employing’Sweeping Gas Membrane Distillation’for Supersaturation Generation, (2015).
